# Power Consumption Analysis of Operating Systems for Wireless Sensor Networks

**DOI:** 10.3390/s100605809

**Published:** 2010-06-08

**Authors:** Rafael Lajara, José Pelegrí-Sebastiá, Juan J. Perez Solano

**Affiliations:** 1 Instituto de Investigación para la Gestión Integrada de Zonas Costeras, Universitat Politècnica Valencia, C. Paranimf, 1, 46730 Gandía, Spain; E-Mail: jolaviz@doctor.upv.es; 2 Instituto de Robótica, Universitat de Valencia, C. Polígono de la Coma, s/n, 46980 Paterna, Spain; E-Mail: juan.j.perez@uv.es

**Keywords:** wireless sensor network operating systems, TinyOS, Mantis, Contiki, MICAz, Tmote

## Abstract

In this paper four wireless sensor network operating systems are compared in terms of power consumption. The analysis takes into account the most common operating systems—TinyOS v1.0, TinyOS v2.0, Mantis and Contiki—running on Tmote Sky and MICAz devices. With the objective of ensuring a fair evaluation, a benchmark composed of four applications has been developed, covering the most typical tasks that a Wireless Sensor Network performs. The results show the instant and average current consumption of the devices during the execution of these applications. The experimental measurements provide a good insight into the power mode in which the device components are running at every moment, and they can be used to compare the performance of different operating systems executing the same tasks.

## 1. Introduction

Wireless Sensor Networks (WSNs) are a very promising technology on which many researchers have focused on their attention. This technology has become a reality thanks to the development of wireless transceivers and microcontrollers with very low power consumption. A wireless transceiver in conjunction with a low power microcontroller, some MicroElectroMechanical Systems (MEMS) to measure physical or chemical variables and a battery are the basic elements that are integrated in the nodes of the network. Due to their small size, low cost and easy deployment, the nodes of the network are usually called *motes*. Motes are small, compact and autonomous devices destined to become ubiquitous. WSN is a technology with an enormous potential that can be used in a high number of heterogeneous applications of interest to society such as environmental monitoring, traffic control, structural monitoring of bridges and buildings, tracking of people and objects, assisted living, *etc*.

The software that runs on the motes plays a fundamental role in the development of WSNs. It controls the mote operation, implements the network protocols and manages the hardware power consumption. Various specific operating systems and programming languages have been proposed to facilitate and speed up the development of new applications. Currently, the most important and widely adopted operating systems for WSN are TinyOS, Contiki and Mantis. The main goals of all of them are to provide a robust and reliable operation and to maintain the mote in the deepest low power mode compatible with the requirements needed at that moment. Low power operation extends the battery lifetime of the motes and it is probably the most important requirement in this type of systems.

A WSN can be considered as an embedded system with severe constraints in terms of memory, computational capacity and power consumption. Traditionally, the development of software for embedded systems with very limited resources has been based on event-driven programming models. TinyOS follows the event-driven model and achieves efficient low power consumption operation and low memory footprint by means of a very simple execution model, similar to the way the hardware works. Contiki is the second operating system being taken into account in this analysis. Contiki, together with TinyOS, are nowadays the most important operating systems for WSN. Both of them support IPv6 in their communications stacks, a key feature for an increasing number of companies and research institutions that are pursuing a seamless connection of WSNs to Internet. Contiki can also be considered an event-driven operating system, but it incorporates programming abstractions to manage the synchronization of concurrent tasks that facilitate the programming of high level sequences of actions. Finally, unlike the first two cases, Mantis is an example of multithreading operating system. The main features of Mantis are the integration of a multithreading scheduler and the programming abstractions that deal with concurrent threads.

The aim of this article is to analyze and compare the low-level current consumption of the mote during the execution of an application running on different operating systems. These measurements reveal the power state of the hardware and the current drawn by the mote during the program execution. Another effect that is evaluated is the noise that the operating system can generate on the supply voltage of the mote due to continuous changes in the hardware power state. This is the case, for example, of a multi-threading scheduler with no task ready to be run. The mote wakes up when the scheduler timer overflows, however since there is no task ready to be executed, the mote immediately goes back to a power down state. This process produces quick transitions in the mote current consumption and fluctuations in its supply voltage. The inconvenience of having this noise affecting the supply voltage is the risk of interfering with sensitive parts of the mote, such as analog sensors.

During the power consumption assessment, a benchmark composed of four applications covering the following operations has been used: scheduling of timed events, data sampling from integrated sensors, data processing and wireless communications.

This paper is divided in the following sections. In Section 2 some related papers and their results are presented. Section 3 is devoted to present the most important features of the four WSN operating systems used in this analysis. The motes that have been used and their comparison with other motes are shown in Section 4. In Section 5 the specification of the applications that have been deployed on each operating system is provided. Section 6 illustrates the average and the instant current drawn by the mote and therefore its power mode during the execution of each task. In Section 7 the final conclusions are provided.

## 2. Related Works

In the bibliography there are a large number of articles concerning new protocols, algorithms and operating systems for WSNs. For example, in Table 1, a list of operating systems proposed in this area is shown. This is a large list considering that TinyOS, which can be considered the pioneer of this type of systems, only dates from the beginning of the last decade. Each one of these operating systems has been developed pursuing different objectives and they present different features. Thus, choosing the most appropriate operating system for one specific application is not an easy matter, because there are a lot of proposals and very few papers with practical evaluations and comparisons between them. In particular, power management can be considered as the most important restriction that developers usually face when they are trying to deploy a real WSN. Consequently, the results that are provided in this article try to compare the real power consumption of the most important operating systems running on typical motes, with the intention of helping developers in their choice.

Until now, there are very few published articles that include assessments and comparisons between different operating systems in terms of power consumption. The first one is a paper in which a comparison between TinyOS and MantisOS is provided [1]. The main contribution of that paper is the evaluation of the performance of event-driven *vs.* multi-threaded systems in terms of power consumption and execution time, but the evaluation does not take into account the interaction between hardware and software and how the operating systems manages the different parts of the mote using power adjustment handlers. On the other hand, article [2] gives an assessment of the battery life of the mote running some applications on different operating systems, but it does not give any information about the instant current drained and its relation with the power state of the mote. Finally, in [3] its authors identify and measure the cost of elementary operations with respect to the overall power consumption, but they do not relate this information with real operating systems. Apart from measuring the average and instant current consumption, in that article, the noise that the operating system can introduce in the power supply of the mote during its operation is taken into account. This is an important matter because the noise can affect the data acquired from the mote’s analog sensors and it has not been considered before in this context. Other authors have confirmed this risk [4] and its effect over sensors has been studied in [5].

## 3. Operating Systems

This section provides a summary of the most important operating systems for wireless sensor networks. The attention has been focused on four of them, basing this selection on certain parameters, such as: the number of publications about them or the activity of the communities that support them. Concerning the number of publications, the percentage of articles related to each operating system included in the main scientific and engineering online databases has been calculated. The databases considered were: IEEE Xplore, ACM Digital Library and Science Direct. The percentages are: 81% TinyOS, 9% Contiki, 8% Mantis and 1% others. The supporting most active communities are the TinyOS development group, with more than 10 new releases in a decade, support for 12 different platforms and an annual TinyOS technology exchange developer meeting, and the Contiki group, with seven releases and a development team composed of people from prestigious companies and research institutions. As a result, the following ones have been selected as the most active and widely accepted: TinyOS Version 1.0 [7,8], TinyOS Version 2.0 [9], Contiki [13] and Mantis [10,11]. At the beginning of this analysis, the SOS [12] operating system was also included as well. But, it was finally discarded due to several problems to make all its modules fully functional and the announcement that it is no longer going to be supported by its developers,

### 3.1. Tinyos version 1.0 (T1)

TinyOS was the first event-driven operating system specific for WSN. It was conceived at the University of California (Berkley) as a collection of components that implement basic operations and it is written in a variant of the C programming language named NesC. TinyOS is considered as a component based operating system due to this property. Components are connected to each other by means of interfaces. New applications can be quickly programmed combining components connected using their interfaces. There are components at the highest level that implement protocols, hardware abstractions, data structures, services, *etc*. Since TinyOS is open source, that is, programmers can combine and adapt its basic components to implement custom applications. Only the components that are really needed in the application are compiled and included in the final executable file, with a significant reduction of the total amount of the mote memory required.

TinyOS provides a robust and reliable functionality by making use of static memory allocation and a non-preemptive FIFO scheduler. All the concurrency mechanisms implemented are the hardware interrupts associated with their handlers. When an interrupt occurs, the microcontroller jumps immediately to the corresponding event handler, stopping the execution of the current task. In TinyOS there are basically three types of procedures: (1) commands executed immediately after its invocation and conceived to perform some action on the hardware elements of the mote, (2) event handlers that interrupt the execution of commands and tasks after being activated by the hardware, and (3) tasks that are functions executed in a deferred way.

Commands and event-handlers constitute the elements associated to the split-phase execution model that represents the usual way in which programs in TinyOS are structured. When the system attempts to perform an action using some hardware component, first it calls a command that sets the order and immediately ends giving back the control to the system. After the hardware configuration carried out by the command, the mote can be placed in a low power state waiting for the hardware response. The second phase occurs when the interrupt from the hardware is fired and the event handler receives the result from the hardware. This event-driven programming model provides concurrency with low memory overhead and saving energy, since it is adapted to the way in which hardware works. Moreover, the mote components can remain in a low-power mode during periods of inactivity.

The main problem of this methodology is the absence of complex concurrency abstractions which make difficult the implementation of mutual exclusion sections or the access to shared resources. For this reason, code specification is mainly made by using state machines that establish the program flow and synchronize the access to the shared system resources. But this programming abstraction based on state machines does not benefit a rapid development of complex new applications. In addition, the lack of support to deal with concurrent tasks makes very difficult to sequence high-level operations and block conditions between tasks.

### 3.2. Tinyos version 2.0 (T2)

The main difference between TinyOS v1.0 and TinyOS v2.0 from the programmer’s point of view is the appearance in the latter of a new class of abstractions, named generic components. They can be included in different components, but each instantiation is a new different copy independent and private from the rest of them. TinyOS v2 also improves some aspects related to the platform support, reliability of the basic components and data structures. The boot sequence has also been changed and it can be blocked during a certain time to avoid race conditions during the execution of different concurrent tasks.

### 3.3. Contiki

Contiki was developed at the Swedish Institute of Computer Science. As in the case of TinyOS, Contiki can be considered an event-driven operating system but with some particularities that facilitate the development of new applications in which there are several concurrent tasks involved. One of the main contributions of Contiki is the introduction of protothreads. This abstraction allows programmers to block conditions that stop a thread waiting for the activation of an event from another concurrent thread. Protothreads simplify and reduce the number of the state machines needed to implement the sequence of high-level operations. The memory overhead introduced by protothreads is very low because they share the same stack and the thread switching only needs a little rewind of the stack positions. Consequently, protothreads combine the energy efficiency and low memory overhead of event-driven models with blocking conditions semantics and programming simplicity of thread-driven models. Programs in Contiki can be disseminated and executed dynamically. Moreover, the last distributions released include a great variety of communication stacks and protocols such as: uIP, SICSlowpan, Rime, *etc*.

### 3.4. MantisOS (MOS)

The last operating system taken into account in this analysis is Mantis, developed at the University of Colorado. It is a specific operating system for WSNs that facilitates the programming of new applications with a completely different approach. Mantis makes use of a multi-threaded scheduler allowing that a short task, with strict time constrains, interleaves its execution with other long complex tasks. The scheduler implements a round robin service and includes a queue of tasks ready to be executed. Using a timer, the scheduler divides the microprocessor time in slices and assigns them to the queued tasks. During each time slice only one task is selected and executed whereas the rest of tasks remain in the queue. This thread-driven model is usually employed in modern operating systems and it prevents a complex task from blocking the execution of other time-sensitive task during too much time.

However, this ability of accommodate different tasks increases the RAM memory footprint and the energy consumed due to the task preemption. Mantis supports binary and counting semaphores that ease the implementation of blocking structures to access shared resources. It is programmed in standard C language that makes easy the inclusion of software from other systems or communication stacks and its portability to different platforms, both real and simulated. The version used in this article is 1.0 beta.

## 4. Platforms

This section exposes the main features of the two platforms used in the analysis: Tmote Sky [14] and MICAz [15,16]. They can be considered platforms for research and experimentation rather than professional devices, but they have become very popular among the research community due to the great availability of open source software developed for them, adaptability to different scenarios and ease of operation. They are the motes most frequently employed in the implementation of testbeds and are usually the typical platforms used for the validation and assessment of new protocols. The wide acceptance of these platforms in academic and research forums led us to consider them as the best option to carry out this work.

Both motes present a very similar architecture based on a microcontroller together with a wireless transceiver and some sensors for measuring physical variables. The main difference between them is the microcontroller, since the Tmote Sky uses the Texas Instruments MSP430F1611 [19] and MICAz relies on the Atmel Atmega128 [21], but both include the same wireless transceiver: the CC2420 [24] from Texas Instruments. Table 2 summarizes the main features of both motes. A detailed presentation of these features is provided below.

The Tmote Sky platform is also known as Telosb. This duality of names comes from the fact that two companies, Moteiv Corporation and Crossbow, shared the same design and they supplied the same mote under different names. Moteiv Corporation has currently changed its name and it is now called Sentilla. Moreover, the company has discontinued this product and is now focused on the development of energy management systems for data centers. Therefore, the proper name of this platform nowadays should be Telosb since this is currently the name under which it is supplied by Crossbow. In any case, due to historic reasons and since a lot of people still refer to this mote as Tmote, this name will be used in the rest of the paper.

The main components of the Tmote platform are the Texas Instruments MSP430F1611 microcontroller and the Texas Instruments CC2420 wireless transceiver. The MSP430F1611 is a ultra-low-power microcontroller that features 10 kB of RAM and 48 kB of program memory (flash). It is a 16-bit processor with several power-down modes and extremely low sleep-current. The MSP430 has a digitally controlled oscillator (DCO) that implements an internal clock of 8 MHz. The microcontroller can wake up from sleep mode in only 6 μs, which allows a short reaction time after the activation of some event. The MSP430 has eight 12-bit ADC channels of which six are accessible on the Tmote expansion connector. The ADC input ranges from 0 to 3.0 V and the maximum sampling frequency is 200 kHz.

Other peripherals are available, including serial peripheral interfaces (SPI), universal asynchronous receiver/transmitters (UART), timers with capture and compare functionality, 2-port 12-bit digital-to-analog converter (DAC) module, a supply voltage supervisor and a 3-port direct memory access (DMA) controller. On the other hand, the CC2420 radio transceiver implements the IEEE802.15.4 standard wireless communication. It offers reliable wireless communication and power management capabilities with a very low-power consumption. The CC2420 is connected to the TI MSP430 microcontroller through the SPI port. Other peripheral components integrated in the Tmote platform are: the USB connection implemented using the FTDI transceiver, a flash memory of 1 Mbyte of capacity and the Sensirion’s SHT15 digital temperature and humidity sensor. A list of operating systems that support this platform is shown in Table 2.

The second platform being used in this article is the MICAz one. This mote is supplied by Crossbow and the main difference with respect the Tmote platform is the microcontroller. The Atmega128 from Atmel is based on an advanced RISC architecture with instructions of 8-bit that are executed in a single clock cycle. The ATmega128 provides 128 kbytes of Flash, 4 kbytes of EEPROM, 4 kbytes of SRAM, 53 general purpose I/O, four flexible Timer/Counters with compare modes and PWM, two USARTs, a byte oriented Two-wire Serial Interface, an 10-bit ADC with 8-channel, a SPI serial port and an internal calibrated RC oscillator. Jointly with the main board of the mote, Crossbow sells sensor boards that can be connected to the MICAz expansion connector, including a great variety of sensors such as: light, temperature, barometric pressure, acceleration/seismic, acoustic, magnetic *etc*. A summary of the mote characteristics and the operating systems with support for this mote is shown in Table 2.

## 5. Applications

The final objective of this article is to compare the performance of the previously mentioned operating systems, providing at the same time results that could be easily reproducible by other researchers. As a first step, the possibility of using typical applications included as examples in the distributions of these operating systems was considered. However, an identical group of applications that were implemented beforehand in all of them could not be found. So, the decision was to conceive a new benchmark composed of four applications performing the most typical actions that the nodes of the network carry out. The same four applications were programmed on each operating system to ensure a fair comparison.

The first one is a program that does nothing and it is called *blank*. With this program, the way in which the operating system manages the power consumption when there is no task to be executed, is evaluated. Since there are some operating systems that do not perform a direct control of the power state of the hardware, a second blank application, named *blank2*, has been programmed, where the code explicitly optimizes the power mode using some management functions, e.g., in TinyOS the components HPLPowerManagementM and McuSleepC have been used, whereas in MOS the impact of the USB interface integrated in the Tmote Sky has been eliminated. This test is particularly relevant because WSN applications require a low power operation.

The second program is the typical *blink* application. With this program, the way the operating systems behave when they have to do a simple task can be determined. In this case, the task constantly changes the state of one LED after a period of time.

With the third program called *xtea* the opposite case is checked, this is, the objective is to evaluate an application that involves the processing of a large quantity of data. To this end, it has been programmed an application in which 32 bits data is coded using the XTEA algorithm [20] in a loop repeated 150,000 times. In addition, the multithreading capabilities of each operating system are tested interleaving the XTEA algorithm with a second task that blinks one LED.

Finally, the last program *sens* performs a typical WSN application. In this case, the microcontroller reads a temperature sensor every second and transmits this value wirelessly. As in the first case, another version of this program has been developed, called *sens2*, with an explicit management of the mote’s power consumption. It should be noticed that different sensors are integrated in each platform and this can be reflected in the final results: MICAz uses a 10 kΩ thermistor whereas Tmote Sky includes the SHT15 sensor from the Sensirion Company.

Contiki and MOS kernels have integrated handlers to control automatically the power consumption of the mote, avoiding the requirement of adding explicit calls to low-power functions in the code to change the hardware state during the inactivity periods. Thus, in these two operating systems, *blank2* and *sens2* applications, that explicitly perform the power management of the mote, are not relevant. However, MOS is not able to automatically control the wireless transceiver and it does not configure properly one of the control lines of the USB interface in the Tmote Sky mote. Therefore, a specific application *blank2* has been developed in MOS, only for the Tmote Sky platform, with a correct configuration of this line for the USB transceiver and another application *sens2* that changes the state of the wireless transceiver during the inactivity periods.

## 6. Results and Disscusion

### 6.1. Experimental setup

First of all, the measuring process of the mote power consumption is presented. Since the supply voltage of the mote is kept constant, the power consumption is directly related to the current drawn. Consequently, the current gives an indication of the total power consumption and it can be measured easily, for example by measuring the voltage drop across a shunt resistor connected in series with the power supply of the mote. There are other articles in which the power consumption is evaluated using the lifetime of a mote powered by batteries. For this purpose, the mote is equipped initially with fully charged batteries and the parameter that is measured is the time period in which the mote remains in operation [2].

In this article, another method to evaluate the mote power consumption has been used. The two premises were to measure the instantaneous consumption, thus ruling out the method of the batteries (as well as there may be many factors that can affect measures), and to achieve a high accuracy. The experimental setup being used is based on a SourceMeter that can generate the 3 V supply voltage and can measure accurately the current supplied. Additionally, a LabVIEW program that communicates with the SourceMeter through a GPIB link to set it up with the supply voltage and the sampling frequency required has been developed. Once the SourceMeter starts the measurement process, it can save the samples in its internal memory until reaching its maximum capacity of 2,500 samples. When the internal memory is full, the SourceMeter sends the data to the PC that represents it on a graph, calculates the mean and variance and saves all this information in a file.

The advantage of this method is the high accuracy of the results obtained. On the other hand, the major limitation is the low sampling frequency that the SourceMeter admits. However, this sampling frequency was enough for the purposes in most of the tests. There was only one case in which a higher sampling frequency was required. For this test, the measurement method was changed and a shunt resistor followed by an amplifier [18] connected to an oscilloscope was used to determine the voltage drop and the current drawn.

### 6.2. Results and discussion for power consumption measurement

The results are focused on the measurement of the instant and average current consumption of the motes running the programs presented in Section 5. The programs were compiled for the MICAz and Tmote Sky platforms, with the total size of the final executable files being the ones shown in Table 3. Regarding the information contained in this Table, it should be noticed that not all the applications could be compiled for all the platforms. Thus, none of the Contiki programs could be compiled for the MICAz platform because by the time this comparison was done, Contiki did not support MICAz. Moreover, in the case of MOS it was not necessary to program the application *Blank2* for the MICAz because MOS does not require an explicit call to the power management functions for this platform. The same condition occurs in Contiki for the Tmote platform and the *Blank2* application.

**Figure 1.**
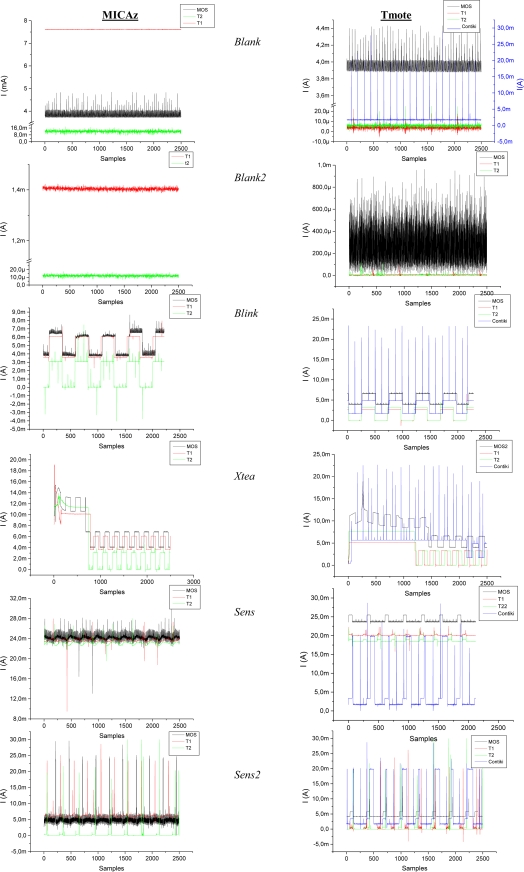
Instant current consumption of each application for both motes.

**Figure 2.**
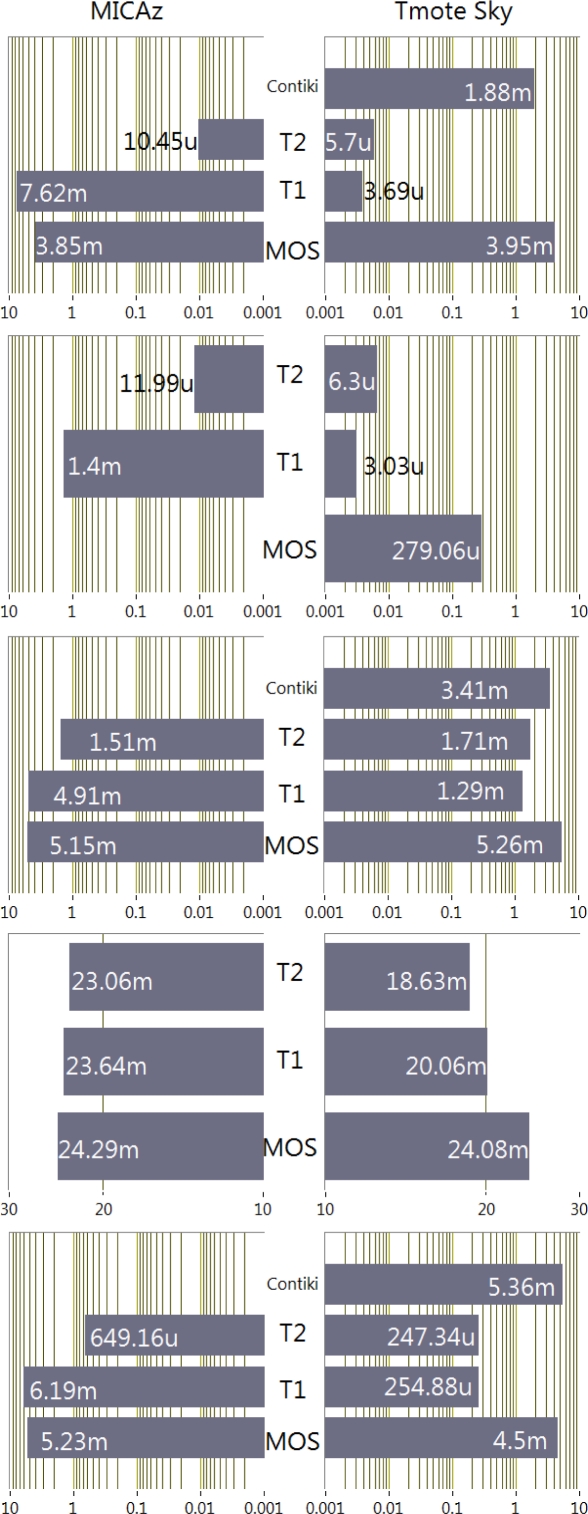
Average current consumption. First row *blank* program; second row *Blank2* program with optimized consumption; third row *Blink*; fourth *Sens* sensing and transmission; fifth row *Sens2* optimized sensing and transmission.

The instant currents drawn by the motes running the test applications are shown in the graphs of Figure 1, whereas the average current is represented in Figure 2. With these graphs the power state of the platform components during the execution of each program can be determined.

The *blank* program results show that T2 has a current consumption very similar for both platforms. The consumption of T2 is the best in the case of MICAz and very close to T1 in the case of Tmote. Most striking is that T1 for MICAz presents a current consumption much higher than the Tmote Sky case. This is due to the different ways in which each operating system manages the microcontroller power modes. For example, the ATmega128 can only be placed in a low-power mode when it is commanded explicitly by means of the adjustPower function. However, in the MSP430 case, the scheduler constantly calculates the lowest power mode that is compatible with the software operation [17]. For its part, MOS estimates the low-power state whenever the scheduler has no tasks to run. The problem in this case is that the microcontroller cannot enter the lowest power down mode, because MOS always needs to leave at least a timer running to manage the scheduler operation. Contiki presents a similar behavior and its efficiency is also worse than TinyOS.

In the program *blank2* the power consumption has been explicitly controlled by calling the power state handler provided by each operating system. Curiously, in T2 the overload introduced by this feature makes that the average current rises, although not very significantly. The same behavior can be seen in T1 running on the Tmote Sky. Nevertheless, T1 on MICAz appreciably reduces the consumption because the microcontroller no longer remains in active mode during the inactivity periods. A great improvement has also been observed in MOS running on the Tmote platform when the line that controls the USB connection is turned off, as indicated in the datasheet [18,19]. This reduction represents about 3.5 mA of the total average consumption.

As it can be seen in Figure 1, the *Blink* application has basically two power consumption levels. One of them corresponds to the *blank* application level and the other one is equal to this level but adding the LED consumption through a series 470 Ω resistor.

The results of the *XTEA* program reveal that MOS is the only operating system that really performs an interleaved execution of two tasks at the same time. The MOS multithreading scheduler can effectively execute several tasks in parallel without the programmer’s awareness. For the rest of operating systems the execution of the two tasks is sequential: first the mote runs the XTEA algorithm and after its completion the *Blink* program. This fact is reflected in the instant current graphs that can be seen in the Figure 1. For T1, T2 and Contiki there are two parts that can be easily distinguished in the graph: the first part is flat and represents the execution of the XTEA algorithm and the second part with a square form that corresponds to the blinking process. Using this program the processing time of two motes can be easily compared. The difference in the execution time can be determined by the number of samples in the instant current graph that the initial execution of the XTEA algorithm takes. Consequently, as it can be seen in the graph, the execution of this algorithm on the MSP430 takes longer than on the ATmega128. Since both microcontrollers have the same clock frequency, the explanation of this result should be found in their internal architectures. Atmel implements an 8-bit Harvard architecture whereas Texas is based on a 16-bit von Newman organization, but above all, the main difference is that Atmel128 can execute an instruction in one clock cycle, whereas MSP430 executes an instruction within a variable interval from 1 to 6 cycles.

In the case of the *Sens* application, the current consumption rises to 23 mA in MICAz, and between 18 and 24 mA in the Tmote Sky. This increment is related to the power mode in which the microcontroller and the transceiver are configured. *Sens* does not explicitly call the power management functions provided by the operating systems and consequently the mote remains in an active mode all the time. So, according to Table 2 (without taking into account the microcontroller clock frequency) the current should be: 500 μA + 17.4 mA = 18 mA for the Tmote Sky and 5.5 mA + 17.4 mA = 23 mA for the MICAz. As it can be seen the average current for the three operating systems approach the theoretical prediction for MICAz: 23,06 mA with T2, 23.64 mA with T1 and 24.29 mA with MOS. Nevertheless, in the Tmote Sky only T2 fulfils the theoretical value with 18.63 mA, whereas T1 and MOS are 1.5 and 3.5 mA respectively above the theoretical expectation. The increment in MOS could be accounted for the 3.5 mA consumed by the USB interface.

Finally, the last program *sens2* drastically reduces the total consumption, especially in T2. *Sens2* include the same power management handlers used in *blank2* program. Contiki and MOS show again the highest consumption, although the latter running on the Tmote Sky could reduce its current disabling the USB. It should be noticed that for Contiki this modification does not improve the results of the previous case, since this operating system handles by itself the activation of the mote components.

### 6.3. Results and discussion for noise measurement

It is important to point out the noise that each operating system introduces in the power supply of the mote due to quick changes in the power state of some hardware elements. For this purpose, the noise level added to the supply voltage during the mote operation running the *Blank* and *Blank2* applications has been evaluated. In Table 4, the variance of the current samples during the execution of these programs for each operating system and each mote is shown. For the estimation of these statistics 2,500 samples were taken, and the weighting is set to sample, the confidence interval is the 95.4%. From Table 4 results, it can be deduced that Contiki is the noisiest one, followed by MOS.

As shown in the graphs of Figure 1, MOS produces a noise level added to the steady current that is higher than with other operating systems. With a more detailed observation of these graphs it can be pointed out that there is a pattern that repeats periodically. The first hypothesis was to blame the operating system scheduler. MOS activates a timer which overflows every millisecond, but the scheduler is invoked by default after 20 ms.

**Figure 3.**
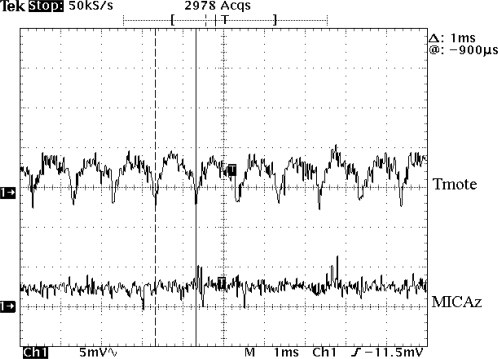
Noise on the supply voltage of the sensors of Tmote Sky & MICAz with MOS. The measurement was made with an oscilloscope; the input channel is AC coupled.

With the measurement system based on the SourceMeter, the sampling frequency was not high enough to determine the source of this noise that could be the timer interrupt, the scheduler or some other element. Therefore, the measurement method had to be changed for the second procedure introduced in the *experimental setup* subsection, which is based on a shunt resistor. This method is much more inaccurate but with this change the sampling frequency could be increased. The result, as shown in Figure 3, is a signal with peaks at intervals of 1 ms. To check whether the noisy signal of 1 ms is related with the main timer of MOS or not, an additional test was carried out changing the overflow period of this timer from 1 ms to 2 ms. The measurements with this new overflow period showed that the noisy signal also changed to 2 ms. So, it can be concluded that the timer operation is what produces this noise. Finally, an important issue is the perturbation that the noise can produce in the sensor’s operation. To evaluate this risk, the conditioning circuit of the analog sensors and its supply voltage was analyzed. The noise affects the supply voltage of the mote and therefore it could perturb the conditioning circuit and the sensor measures. To evaluate of this effect the temperature sensors included in the motes were used. In MICAz, there is a thermistor that is fed by means of a microcontroller output pin instead of through the general power supply signal of the mote. The test that was performed to find out how the noise affects the sensors was focused on monitoring the supply voltage of this sensor. The result of this test is the voltage graph shown in Figure 3.

This plot lets us assert that there is almost no noise present on this line during the sensor operation. The Tmote Sky case is not different because, although the temperature sensor is the digital SHT15 sensor from Sensirion, it is also powered through a microcontroller digital output and the measured noise level is negligible as well. Despite the fact that very low levels of noise in the sensor supply lines were found, this matter should be taken into account during the design of new motes. In the case of sensitive sensors that require a signal conditioning or amplification, filtering the sensors supply line is recommend, even if they are fed from some microcontroller digital pin.

## 7. Conclusions

After the development of all the applications for the different operating systems shown in this article, it can be concluded that programming applications in C implies a much less steep learning curve than when the application is programmed in NesC, such as is the case of TinyOS. In NesC the programmer has to get used to a new programming paradigm that includes concepts such as: components, modules, configurations, interfaces, *etc*. The positive side of the TinyOS programming is the efficiency that can be achieved in terms of code size (see Table 3) and energy consumption.

According to the results presented, in general T2 is more efficient in terms of power consumption than T1, MOS and Contiki. In the case of the Tmote Sky platform the difference between T1 and T2 is minimal, even though T1 is a little more efficient in simple programs. Moreover, in Section 3 it is seen that T2 is simpler than T1, Contiki and MOS. As expected, in terms of energy efficiency, a simple system normally consumes less than a more complex one. The real question is whether this improvement of the power consumption implies to accept lower capabilities for the final system or not. There is no single answer to this question because it depends on whether a particular application is looking for the implementation of advanced features, like parallel execution of complex tasks, or a further optimization of the power consumption. In most applications developed the latter is chosen because WSN is a field in which it very is important to maximize the network lifetime.

## Figures and Tables

**Table 1.** Operating Systems for Wireless Sensor Networks.

**OS**	**Model**	**ROM Memory**	**RAM Memory**	**Type of Processes**
TinyOS v1	Events	3.4 kbytes	336 Bytes	Tasks, commands and event handlers
TinyOS v2	Events	3.4 kbytes	336 Bytes	Tasks, commands and event handlers
Contiki	Events	3.8 kbytes	230 Bytes	Protothreads
MantisOS	Multithreading	14 kbytes	500 Bytes	Threads
Nano-RK	Multithreading	10 kbytes	2,000 Bytes	Tasks with priority
t-kernel	Multithreading	28.2 kbytes	2,000 Bytes	Threads
Bertha	Mobile agents	10 kbytes	1,500 Bytes	Process fragments
CORMO	Events	5.5 kbytes	130 Bytes	Tasks and event handlers
SOS	Events	20 kbytes	1,163 Bytes	Tasks defined as modules
SenOS	State Machines	Not specified		Processes

**Table 2.** Main properties of Tmote Sky (Telosb) and Micaz platforms.

	**Tmote Sky (Telosb)**		**MICAz**
**Microcontroller**	**Texas MSP430 F1611**		**Atmel ATmega128(L)**
Vcc	1.8 .. 3.6 V		2.7 .. 5.5 V
Instant current consumption	Active	500μA @ 1MHz, 3V	Active 5.5mA @ 4MHz, 3V
	Standby	2.6 μA	Power down 5 μA
	Off	0.2 μA	
Wakeup time	6 μs		4.1 ms
Architecture	RISC 16 bits		RISC 8 bits
Flash	48 kB		128 kB
RAM	10 kB		4 kB
EPROM			4 kB
A/D	12 bits, 8 channels		10 bits, 8 channels
D/A	12 bits, 2 channels		
Communications	JTAG, 2xUART, 2xSPI, I2C, 3xDMA		JTAG, 2xUART, SPI, I2C

**Transceiver**	**CC2420**	
Vcc	2.1 … 3.6 V	
Transmission power	0, 5, −10, −15, −25 dBm	
Sensitivity	−95 dBm	
Instant current consumption	RX	18.8 mA
	TX	17.4 mA (@ 0 dBm)	
	sleep	426 μA
	Power down	20 μA
	off	0.02 μA
Startup time	1 ms (xtal oscillator)	
Radio range	Over 120 m outdoors with 0 dBm	

**External Memory**	**ST M25P80**		**AT45DB041B**	
	Flash Memory 1 MB		Flash memory 512 KB	
Vcc	2.7 … 3.6 V		2.5 … 3.6 V	
Instant current consumption	Read	>4 mA	Read	4 mA
	Standby	>50 μA	Standby	2 μA
	Power-down	1 μA		
Interface	SPI		SPI	

Sensors	On board integrated sensors: humidity, temperature and light.	Expansion connector that includes digital I/O, analog inputs, I^2^C, SPI and UART. There are available expansion boards with light, temperature, RH, barometric pressure, accelerometers, acoustic and magnetic.

Operating systems	TinyOS v1, Tinyos v2, Contiki, Mantis OS, Sos, RETOS [23]	TinyOS v1, Tinyos v2, Contiki, Mantis OS, Sos, Nano-RK [22],

**Table 3.** MICAz and Tmote Sky program sizes expressed in B (Bytes) or kB (kbytes).

		**Blank**	**Blank 2**	**Blink**	**XTEA**	**Sens**	**Sens 2**
**MICAz**							

T1	ROM	476 B	620 B	1,674 B	1,790 B	11,402 B	11,594 B
	RAM	19 B	21B	48 B	64 B	441 B	443 B
T2	ROM	680 B	686 B	2,218 B	2,104 B	11,890 B	13,906 B
	RAM	4 B	4 B	51 B	49 B	278 B	331 B
MOS	ROM	26 kB	-	26 kB	27 kB	30 kB	30 kB
	RAM	1 kB	-	1 kB	1 kB	1.1 kB	1.1 kB

**Tmote Sky**							

T1	ROM	1,586 B	1,586 B	2,722 B	2,858 B	13,040 B	13,203
	RAM	27 B	27 B	45 B	45 B	405 B	407 B
T2	ROM	1418 B	1,430 B	2,654 B	2,656 B	12,198 B	14,068 B
	RAM	4 B	4 B	55 B	35 B	328 B	384 B
MOS	ROM	14 kB	15 kB	14 kB	14 kB	16 kB	16 kB
	RAM	1.6 kB	1.6 kB	1.6 kB	1.6 kB	1.7 kB	1.7 kB
Contiki	ROM	20.8 kB	-	20.9 kB	21 kB	21 kB	21 kB
	RAM	2.3 kB	-	2.3 kB	2.3 kB	2.3 kB	2.3 kB

**Table 4.** Variance of the current samples taken.

	**Blank**		**Blank2**	

	**MICAz**	**Tmote Sky**	**MICAz**	**Tmote Sky**
**T1**	1.833E-12	2.06E-12	1.679E-12	63.749E-12
**T2**	20.8E-12	2.872E-12	29.058E-12	11.8E-12
**MOS**	22.2E-9	5.482E-9	-	35.409E-9
**Contiki**	-	3.41E-6	-	-
